# A Case of Nasal Myiasis Without Any Predisposing Factors: An Uncommon Presentation

**DOI:** 10.7759/cureus.106566

**Published:** 2026-04-07

**Authors:** Sara Elaissaoui, Hicham Mimouni, Mohammed Er-Rami, Rajae Borki, Ilham Rkain

**Affiliations:** 1 Department of Otorhinolaryngology-Head and Neck Surgery, University Hospital Mohammed VI, Tangier, MAR; 2 Department of Parasitology and Mycology, Moulay Ismail Military Hospital, Meknes, MAR; 3 Department of Otorhinolaryngology-Head and Neck Surgery, University Hospital Mohammed VI, Faculty of Medicine and Pharmacy of Tangier, Abdelmalek Essaadi University, Tangier, MAR

**Keywords:** case report, ent myiasis, immunocompetent patient, oestrus ovis, rhinosinusitis, tropical diseases

## Abstract

Nasal myiasis is an uncommon human parasitic disease affecting the nasal cavity. In otolaryngology, myiasis is mostly encountered as aural myiasis and oral myiasis, but occurs rarely as nasal myiasis. It typically affects individuals with poor hygiene, chronic illness, or immunosuppression. Cases in immunocompetent individuals are exceptional and can present diagnostic challenges. We report the case of a 32-year-old immunocompetent female who presented with the spontaneous expulsion of small larvae through the nostrils. Live larvae were observed in both nasal cavities during endoscopic examination. Parasitological analysis confirmed the presence of Oestrus ovis larvae. No underlying immunodeficiency or predisposing condition was identified. Imaging revealed bilateral maxillary sinusitis with anterior ethmoiditis. No surgical removal was performed, and the patient received only oral albendazole. To our knowledge, only a few cases of nasal myiasis have been reported in immunocompetent hosts, highlighting the rarity of this presentation. This report underscores the importance of considering nasal myiasis in the differential diagnosis of nasal symptoms, even in immunocompetent individuals. Early recognition and prompt management are essential for preventing complications and ensuring optimal patient outcomes.

## Introduction

Myiasis refers to the infestation of mammalian tissues by the larvae of dipteran flies. The most frequent presentation is cutaneous myiasis, whereas internal organ involvement is relatively uncommon [1]. Nasal myiasis, a parasitic infestation of the nasal cavity by fly larvae, is an infrequent condition that typically occurs in individuals with compromised immunity, poor hygiene, or unfavorable socioeconomic circumstances [2]. In Morocco, species belonging to the Oestridae family are most commonly implicated, although rare cases attributed to Lucilia species from the Calliphoridae family have also been reported [3]. Despite its rarity, rhinomyiasis should be included in the differential diagnosis for travelers returning from tropical or rural Mediterranean areas who present with rhinosinusitis-like symptoms that are unresponsive to standard medical therapy [4]. Management primarily relies on the complete removal of larvae, achieved through manual extraction, endoscopic techniques, or surgical intervention in cases of deep tissue invasion [3].

This case report has been prepared in accordance with the Surgical CAse REport (SCARE) 2025 checklist [5].

## Case presentation

**Figure 1:**
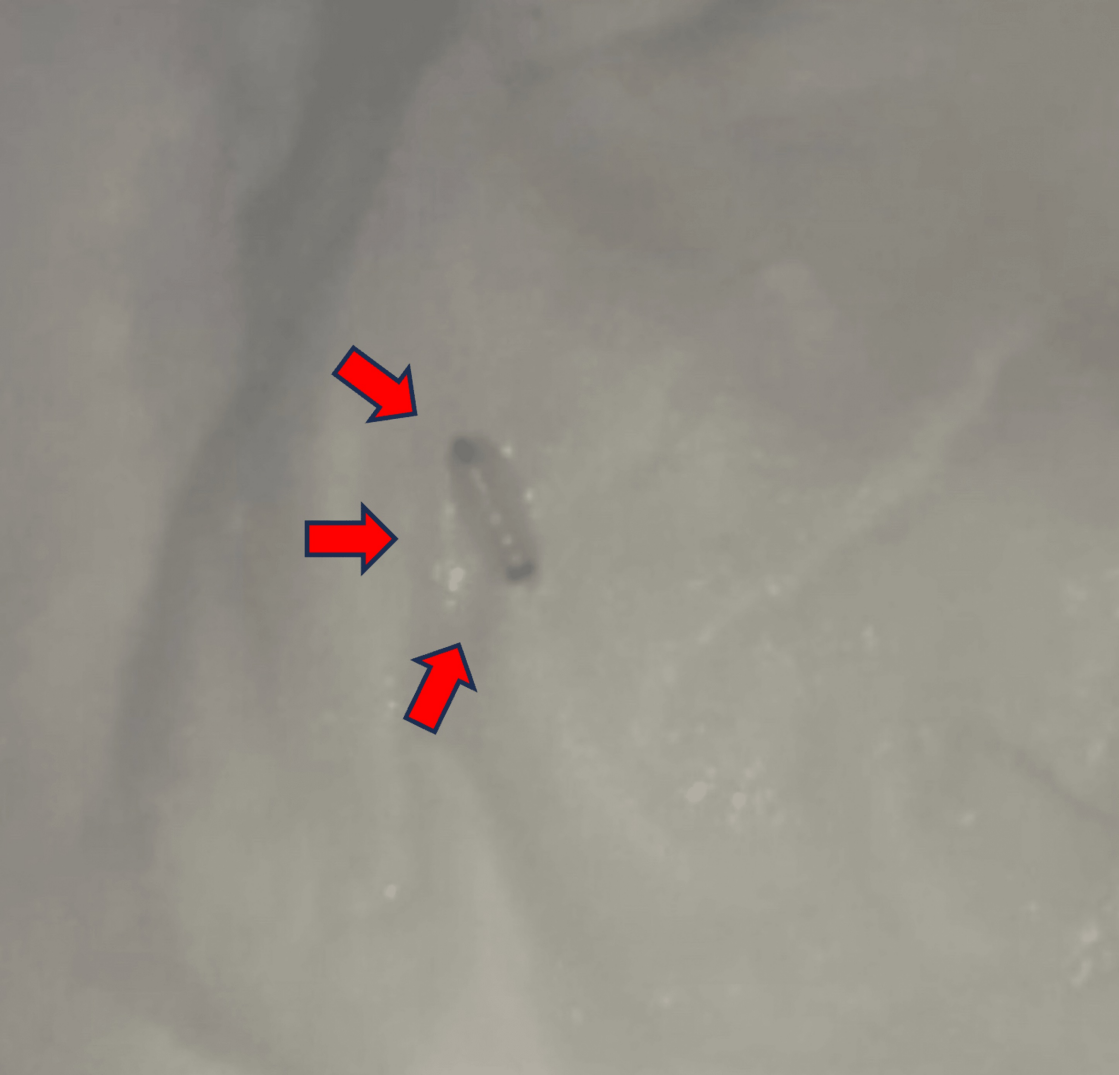
Camera phone image showing a larva (arrows), magnification x10

A 33-year-old female patient with no significant medical history, notably with no travel to tropical areas, no immunosuppression, and no direct contact with animals, presented to the ENT (ear, nose, and throat) emergency department with the spontaneous discharge of small larvae from her nostrils (Figure 1), ongoing for the past three days. The patient’s history revealed an episode of nasopharyngitis two weeks earlier, treated symptomatically, without real improvement. She continued to experience persistent anterior clear, non-purulent, and non-bloody rhinorrhea. She reported no fever, headache, facial pain, or general health deterioration.

**Figure 2:**
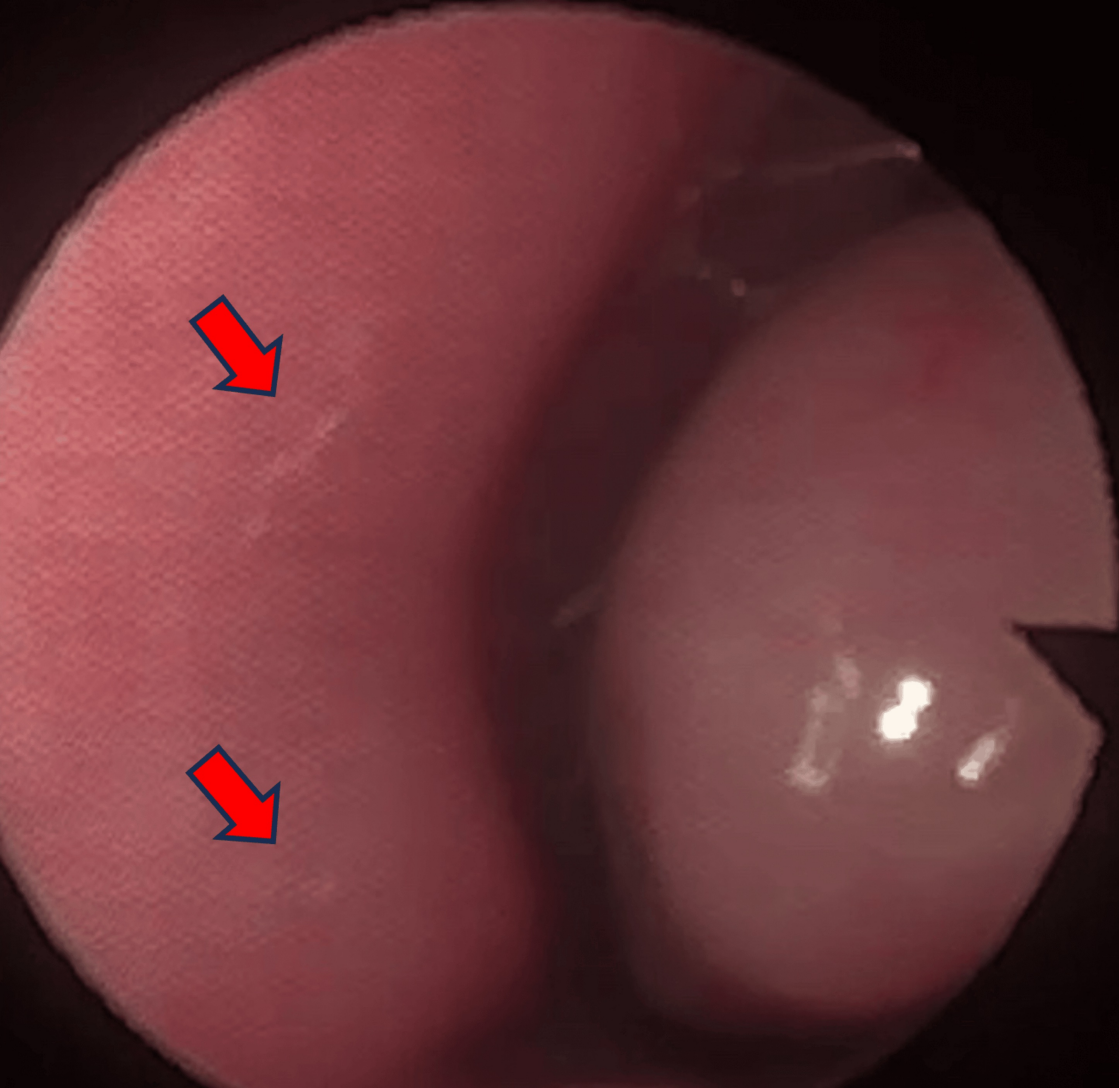
Nasofibroscopy image showing millimetric, mobile photophobic larvae (arrows)

Clinical examination revealed that the patient was in good general condition, with a body temperature of 37 °C, and unobstructed nasal breathing. Nasal endoscopy showed inflamed mucosa and several mobile larvae, present in both nasal cavities and in contact with mucoid secretions, without any obvious evidence of mucosal necrosis, atrophy, or underlying lesions (Figure 2).

**Figure 3:**
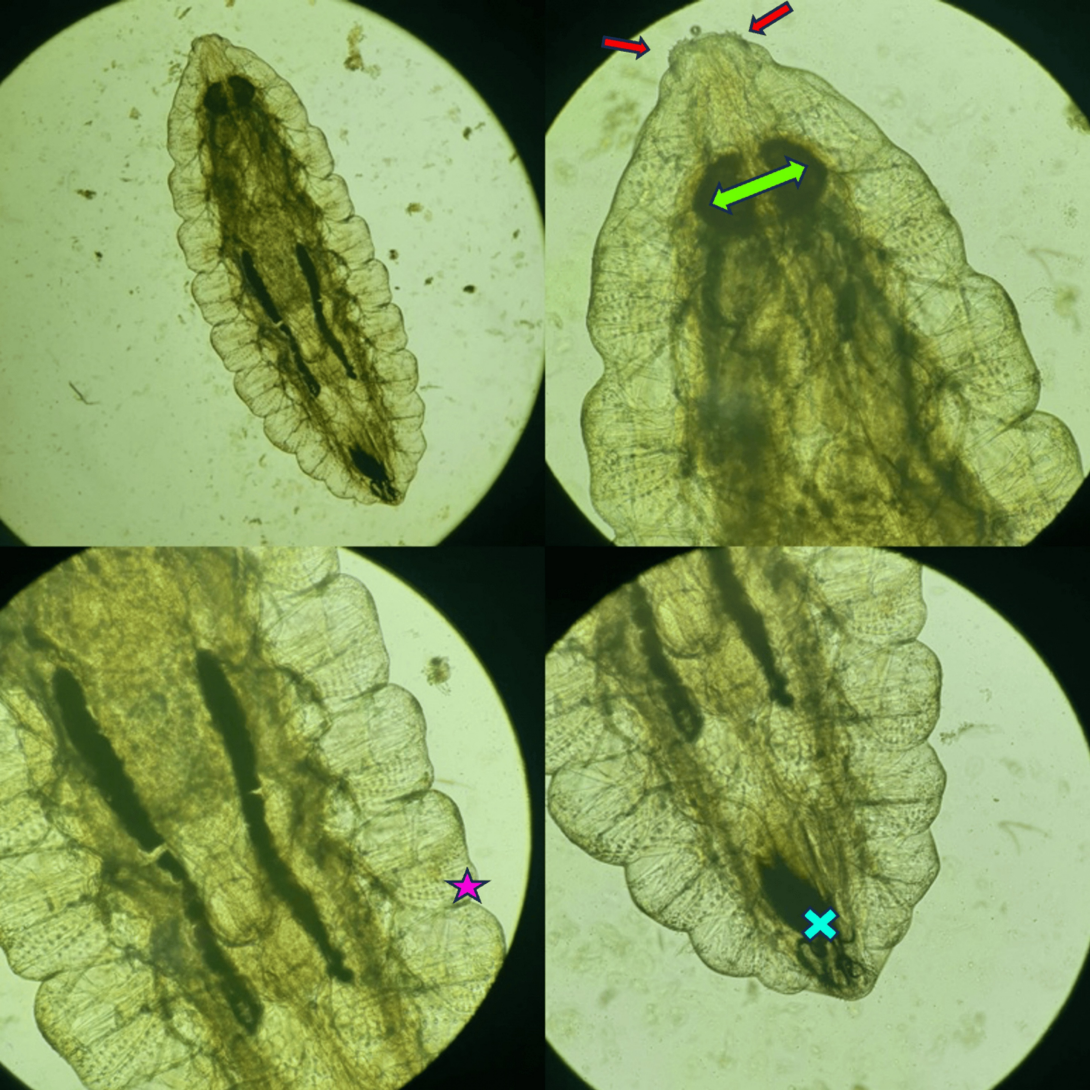
Magnifying lens shots showing an Oestrus Ovis larva with buccal hooks (cross), stigmas (double-headed arrow), rows of spines (star), and spiny hooks (arrows)

A nasal swab was performed for parasitological identification. The observed larvae were tiny (1-2 mm in length), translucent, and had tapered extremities. Macroscopic examination under a magnifying lens revealed a body composed of 12 metameres. The anterior end bore two curved, black oral hooks. The posterior end displayed two respiratory stigmas containing several small, punctiform spiracular openings dispersed within the peritreme. On the ventral surface, each metamere presented four rows of spines. The posterior extremity also exhibited small, thorn-like hooks arranged in two clusters on either side of the midline. All these morphological characteristics were consistent with the identification of the larva as a first-instar (L1) stage of Oestrus ovis (Figure 3). 

**Figure 4:**
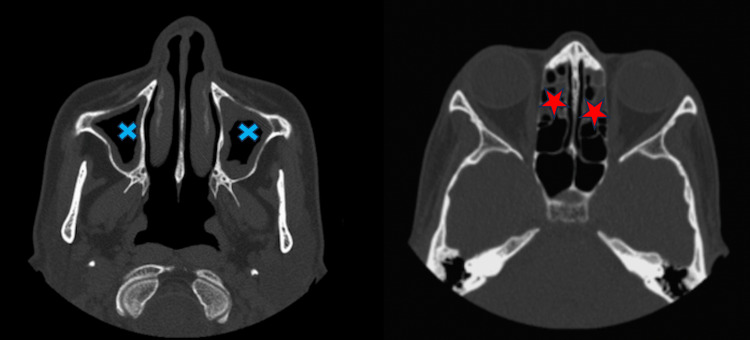
Axial CT images showing maxillary sinus (crosses) and anterior ethmoidal sinus opacification (stars) CT: computed tomography

A copro-parasitological sample showed no eggs, cysts, or vegetative forms of parasites. CT scans of the sinuses revealed mucosal thickening of the maxillary sinuses, with obstruction of the left maxillary ostium and hypodense opacification of several anterior ethmoidal air cells on both sides. The nasal septum appeared normal, with no evidence of bone destruction or lysis (Figures 4A, 4B).

The patient was treated with oral albendazole 400 mg once weekly for two consecutive weeks, along with regular nasal saline irrigation. Subsequent clinical and endoscopic evaluations demonstrated improvement and complete resolution of the infestation, with no recurrence observed during a three-month follow-up period. Because of the favorable clinical response and good therapeutic adherence, mechanical extraction was not necessary.

## Discussion

Myiasis caused by Oestrus ovis, a zoonotic infestation by Diptera larvae typically affecting goats and sheep in tropical and Mediterranean regions, is an unusual occurrence in humans [4]. Oestrus ovis, first described by Linnaeus in 1758, is a dipteran fly that completes three larval stages as a parasite within the sinonasal cavities of sheep and goats during the winter season [6]. When the parasite infests the human nasal cavity, it may cause tissue destruction within the nasal passages and paranasal sinuses. The migration of larvae can extend the damage to neighboring structures, including the eyes and the cranial cavity, potentially resulting in mucosal necrosis, atrophy, or even bony erosion [7].

Several factors have been reported to predispose individuals to myiasis, including overcrowding, poor socioeconomic and sanitary conditions, inadequate personal hygiene, immunosuppression, chronic illnesses (such as diabetes mellitus), malignancy, open wounds, infections, prolonged debility, tube feeding, psychiatric disorders, and hemiplegia [1]. Interestingly, no predisposing factor was identified in our patient. Nasal myiasis is more commonly encountered in tropical regions and is often associated with chronic sinonasal disorders or systemic comorbidities. Nevertheless, cases occurring in otherwise healthy individuals are rare [2]. Although uncommon, rhinomyiasis should be considered in patients returning from tropical or rural Mediterranean areas who present with rhinosinusitis-like symptoms unresponsive to standard treatments [4].

The diagnosis relies primarily on clinical history and physical examination. In our patient, parasitological findings were essential to confirm the diagnosis. The typical presentation includes epistaxis, thick mucopurulent nasal discharge, nasal blockage with foul odor, facial pain, headache, and occasionally the sensation of a moving foreign body within the nasal cavity [8]. Patients who report a crawling sensation in the nose or sinuses may indeed have sinonasal infestation by the sheep nasal bot fly, as several human cases have previously been reported [6]. In tropical environments, nasal myiasis may complicate allergic rhinitis. Therefore, early recognition and preventive measures, such as regular nasal douching and the use of mosquito nets during sleep, can reduce the risk of serious complications like palatal perforation or intracranial invasion [9].

Management of nasal myiasis generally involves manual removal of larvae, nasal irrigation with saline, and systemic broad-spectrum antibiotics, which are considered effective and practical in low-resource settings [10]. Because larvae can rapidly invade adjacent tissues, early intervention is crucial. Reported treatments include topical turpentine application, endoscopic extraction, and close follow-up [8]. In our case, early diagnosis allowed successful nonsurgical management with oral albendazole alone. Although myiasis remains a rare condition, maintaining proper hygiene, prompt diagnosis, and timely therapy are essential to prevent severe complications, especially in individuals with risk factors such as immunosuppression, poor hygiene, malnutrition, diabetes, or vascular disease [11].

## Conclusions

This report emphasizes that nasal myiasis may occur even in immunocompetent patients, an exceptionally rare presentation. Timely diagnosis and appropriate pharmacologic treatment can lead to complete resolution without the need for surgical intervention. Awareness of this possibility is important, and clinicians should include it in the differential diagnosis of persistent or unexplained nasal complaints.
